# SEOM guide to primary and secondary prevention of cancer: 2014

**DOI:** 10.1007/s12094-014-1215-5

**Published:** 2014-10-31

**Authors:** P. P. Segura, J. P. B. Fombella, B. P. Lorenzo, M. R. Martín, P. G. Lopez

**Affiliations:** 1 Medical Oncology Department, Hospital Clínico San Carlos, Madrid, Spain; 2 Medical Oncology Department, Hospital Universitario Central de Asturias, Oviedo, Spain; 3 Medical Oncology Department, Hospital Universitario de Valladolid, Valladolid, Spain; 4 Medical Oncology Department, Hospital General Río Carrión, Palencia, Spain; 5 Medical Oncology Department, Hospital Ramón y Cajal, Madrid, Spain

**Keywords:** Prevention, Primary and secondary, Cancer, SEOM

## Abstract

**Purpose:**

The current incidence of cancer in the world is 14 million cases in 2012, with a mortality rate of 8.2 million in that year. The incidence of cancer in Spain exceeds 215,000 cases a year, and survival rates are the highest when compared to those of our neighbouring countries. Among the reasons for the steady decrease in cancer mortality rates in Spain, two causes must be highlighted: the increasing efficacy of treatment and prevention measures. It is important evaluate the opportunity of early detection and prevention in these tumors.

**Methods:**

We have reviewed the evidence published in the most prevalent tumors. The evidence levels described in this paper are based on the GRADE system.

**Results:**

We show the recommendations about primary and secondary prevention in breast cancer, cervical cancer, colorectal cancer, prostate cancer and lung cancer.

**Conclusion:**

The diffusion of these preventive tools can reduce the incidence of cancer and increase the number of early diagnostics in the most prevalent tumors.

## Introduction

The current incidence of cancer in the world is 14 million cases in 2012, with a mortality rate of 8.2 million in that year [1]. The incidence of cancer in Spain exceeds 215,000 cases a year, and survival rates are the highest when compared to those of our neighbouring countries. Among the reasons for the steady decrease in cancer mortality rates in Spain, two causes must be highlighted: the increasing efficacy of treatment and prevention measures.

The present study analyses tumours in which prevention has shown to be of use, or is at least being debated as a useful possibility. Both primary and secondary prevention measures have been assessed.

The evidence levels described in this paper are based on the GRADE system (Table 1).

**Table 1:** GRADE system classification of the quality and strength of evidence

	Code
Quality of evidence	
High	A
Moderate	B
Low	C
Very low	D
Strength of evidence	
Strong for/against an intervention	1
Weak for/against an intervention	2

Grade of recommendation	Risk/benefit	Quality of evidence	Implications
1A Strong recommendation High-quality evidence	Benefits clearly outweigh risk and burdens	Consistent evidence from well-performed randomised controlled trials or overwhelming evidence of some other form. Further research is unlikely to change our confidence in the estimate of benefit and risk	Strong recommendation Can apply to most patients in most circumstances without reservation
1B Strong recommendation Moderate-quality evidence	Benefits clearly outweigh risk and burdens	Evidence from randomised controlled trials with important limitations, or very strong evidence of some other form. Further research is likely to have an impact on our confidence in the estimate of benefit and risk and may change the estimate	Strong recommendation Likely to apply to most patients
1C Strong recommendation Low-quality evidence	Benefits appear to outweigh risk and burdens	Evidence from observational studies, unsystematic clinical experience, or from randomised, controlled trials with serious flaws. Any estimate of effect is uncertain	Relatively strong recommendation Might change when higher quality evidence becomes available
2A Weak recommendation High-quality evidence	Benefits closely balanced with risks and burdens	Consistent evidence from well-performed randomised, controlled trials or overwhelming evidence of some other form. Further research is unlikely to change our confidence in the estimate of benefit and risk	Weak recommendation Best action may differ depending on circumstances or patients or societal values
2B Weak recommendation Moderate-quality evidence	Benefits closely balanced with risks and burdens, some uncertainities in the estimates of benefits, risks and burdens	Evidence from randomised, controlled trials with important limitations, or very strong evidence of some other form. Further research is likely to have an impact on our confidence in the estimate of benefit and risk and may change the estimate	Weak recommendation Alternative approaches likely to be better for some patients under some circumstances
2C Weak recommendation Low-quality evidence	Uncertainty in the estimates of benefits, risks, and burdens; benefits may be closely balanced with risks and burdens	Evidence from observational studies, unsystematic clinical experience, or from randomised, controlled trials with serious flaws. Any estimate of effect is uncertain	Very weak recommendation Other alternatives may be equally reasonable

## Breast cancer

Breast cancer (BC) is the most frequent cancer among women and the first cause of death among European women. In 2012, the estimated age-adjusted annual incidence in the European Union was 82.1/100,000 and the mortality 15.5/100,000 (GLOBOCAN). The incidence has increased after the introduction of mammography screening and the ageing of the population and the mortality has decreased because of improved treatments and early detection. The result of this trend is a longer survival rate.

The focus of this guideline is summarising the current evidence on prevention of BC in women at average risk of developing BC. In this subgroup, women must have no symptoms, no history of invasive BC, ductal or lobular carcinoma in situ or atypical hyperplasia, no family history in a first-degree relative, no suggestion/evidence of a hereditary syndrome and no history of chest wall radiation. For women at increased risk, please consult the SEOM clinical guideline for hereditary cancer.

### Primary prevention

The main risk factors for BC (personal, familiar and reproductive factors and age) cannot be changed. Regular physical activity beginning in the early childhood, weight control and alcohol intake reduction are strategies that can decrease the probability to develop this disease. The use of pharmacologic interventions for BC reduction is not recommended for women at average risk.

### Secondary prevention

Early detection through screening remains the primary tool available to healthy women in preventing the development of BC, and thus reducing BC mortality.

#### Mammography

Screening mammography is currently the best available method to detect BC at an early stage and the only that is associated with significant reduction in mortality [3]. Expert groups and medical societies recommend routine screening with mammography for women aged 50 and older [4–6]. However, there is controversy about women in their 40s, due to lower risk of BC, smaller reduction in mortality compared with older women and higher impact of harms related to screening mammography (false-positive and overdiagnosis rates) [7]. The decision to start screening before age 50 years should be an individual one and should take into account patient context, including the patient’s values regarding specific benefits and harms. There is insufficient current evidence to assess the additional benefits and harms of screening mammography in women aged 75 years or older. The appropriate interval for screening mammography is 2 years (Table 2).

**Table 2:** Screening recommendations for BC in women at average risk

Population	Women aged 40–49	Women aged 50–69	Women aged 70–74
Mammography	Individualise, every 2 years 2C	**Routinely, every 2** **years** 2B	**Routinely, every 2** **years** 2C
MRI	Not recommended 2D	Not recommended 2D	Not recommended 2D
CBE	Not recommended 2C	Not recommended 2C	Not recommended 2C
BSE	Not recommended 2B	Not recommended 2B	Not recommended 2B

Words in bold identify the main recommendations

#### Magnetic resonance imaging (MRI)

There are no data evaluating whether screening women at average risk of breast cancer using MRI scans reduce mortality as compared with mammography or no screening.

#### Breast examinations

No evidence is found to show that clinical breast examination (CBE) or breast self-examination (BSE) reduces the risk of mortality.

BC screening remains a subject of intense debate. Although mammography remains the gold standard, it also has limitations and recent studies raise questions of potential harms [3]. Besides, as treatment of clinically detected disease improves, the benefit of the screening probably diminishes. Optimal BC screening will probably require a personalised approach, with application of technologies best suited to the individual`s age and risk.

## Cervical cancer

### Primary prevention

The most important cause of cervical cancer is persistent human papillomavirus (HPV) infection, in particular the oncogenic subtypes such as HPV 16 and 18. Randomised clinical trials have shown that HPV 16 and 18 vaccination is highly effective in preventing moderate-grade cervical dysplasia or worse pathology (CIN2+) among women not previously exposed to these types of HPV [8, 9]. There currently are 2 HPV vaccines available: a quadrivalent vaccine that protects against infection by HPV types 6, 11, 16 and 18 and a bivalent vaccine that protects against HPV types 16 and 18. These vaccines will be more cost-effective if given prior to initiating sexual activity, and the priority target population is young adolescent females

### Secondary prevention

The Papanicolaou (Pap) smear is the method of choice in screening for cervical cancer. In the last years testing for HPV has been proposed as an adjunct or alternative to Pap test screening. Both have been compared in several large trials and the findings do not suggest a clearly dominant screening strategy. Cervical cancer screening should begin at age 21 years; women should discontinue screening after age 65 years if they have no history of CIN2+ within the last 20 years and have had adequate negative prior screening (3 consecutive negative cytology tests or 2 consecutive negative co-tests within the previous 10 years, with the most recent test occurring within the last 5 years)

#### Recommendations

- Routine HPV vaccination is recommended mainly for females aged 11–12 years, but also for females aged 13–18 to catch up those who have not been previously vaccinated or completed their vaccine series (grade 1A). Standard screening for cervical cancer should continue in vaccinated women.
- For women aged 21–29 years, screening with cytology alone every 3 years is recommended (grade 1B). HPV testing should not be used to screen women in this age group.
- Women aged 30–65 years should be screened with the combination of cytology and HPV testing every 5 years, but it is also acceptable the screening with cytology alone every 3 years (grade 1A).
- Women who have undergone total hysterectomy with removal of the cervix and have no history of CIN2+ should not be screened for cervical cancer (grade 1B) (Table 3).

**Table 3:** Recommendations for the screening of cervical and prostate cancer

Cancer site	Population	Recommended screening method
Cervix (women)	Aged <21 years	No screening
	Aged 21–29 years	Cytology alone every 3 years
	Aged 30–65 years	HPV and cytology every 5 years (preferred) or Cytology alone every 3 years (acceptable)
	Aged >65 years	No screening if adequate negative prior screening and no history of CIN2+
	After hysterectomy	No screening
Prostate (men)	Aged 50–70 years	PSA ± DRE
	PSA <3 ng/mL	Repeat every 1–2 years
	PSA 3–4 ng/mL	Individualised risk assessment
	PSA ≥4 ng/mL	Consider biopsy or repeat test in 6–12 m

*HPV* human papillomavirus, *CIN2* cervical intraepithelial neoplasia grade 2, *PSA* serum prostate-specific antigen, *DRE* digital rectal examination

## Prostate cancer

### Primary prevention

There is strong evidence indicating an association between androgens and the risk of prostate cancer. The 5α-reductase inhibitors that are used to treat benign prostatic hyperplasia block the conversion of testosterone to dihydrotestosterone and have shown to reduce the risk of prostate cancer [10]. Moreover, several randomised trials using antioxidants such as selenium and vitamin E have failed to demonstrate benefit.

### Secondary prevention

Currently, best evidence supports the use of serum prostate-specific antigen (PSA) for the early detection of prostate cancer. However, many experts continue to recommend digital rectal examination (DRE) for screening, as some clinically significant cancers may potentially be missed using a serum PSA cut point alone. The true benefits of prostate cancer screening are unclear despite the results from two large randomised trials [11, 12] investigating whether PSA testing reduces prostate cancer mortality. In addition, early detection results in identification of many men with indolent disease that could not benefit from immediate treatment. False positive results of PSA and DRE can contribute to patient anxiety, increased costs and potential complications associated with unnecessary biopsies. For these reasons, the patient and the physician should discuss the risks and potential benefits prior to testing.

#### Recommendations

- Asymptomatic men with a PSA <3.0 ng/mL who are regularly screened with PSA or are anticipating undergoing annual PSA screening for early detection of prostate cancer may benefit from a discussion of both the risks and benefits of 5α-reductase inhibitors (grade 2B).
- Screening is recommended with PSA with or without DRE for well-informed men aged 50–70 years (grade 1A).
- For men with PSA <3 ng/mL, the test should be repeated every 1–2 years (grade 1B).
- For men whose PSA is >4 ng/mL or those with DRE suspicious for cancer at any PSA level, a biopsy should be considered. If not biopsy is performed, repeat testing in 6–12 months (grade 1B). Percent-free PSA may be assessed in selected patients with PSA values between 4 and 10 ng/mL.
- For PSA levels between 3 and 4 ng/mL, physicians should consider an individualised risk assessment that incorporates other risk factors. These factors include age, family history, ethnicity, DRE or PSA kinetics (grade 2C) Table 3.

## Colorectal cancer

Colorectal cancer (CRC) is the most frequent type of cancer among the population. Mortality rates have decreased in past years, mainly as a result of the implementation of CRC screening programmes in many industrialised countries.

The different known aspects of primary and secondary prevention, as well as the evidence levels described in the scientific literature, are discussed in the following paragraphs.

## Primary prevention [13–16]

- Consumption of red meat, processed meat and cooked meat that is very well done or has been prepared in direct contact with the source of heat should be moderate (Grade B recommendation).
- A low-fat diet that is high in fibre, fruit and vegetables is advisable (Grade B), although published results are not conclusive. A diet that is high in milk and dairy products is also recommended (Grade B).
- Folate, calcium and vitamin D intake must be adequate (Grade B), but not provided in the form of dietary supplements.
- Antioxidant supplements should not be taken.
- Physical exercise and avoidance of excess weight and obesity must be encouraged to prevent CRC (Grade B).
- NSAIDs and aspirin should not be taken systematically to prevent CRC (Grade B), nor should hormone treatment be administered (Grade A).

### CRC screening recommendations in the average risk population [15] (Table 4)

Individuals of 50 years of age and over, with no additional risk factors, are considered to be at average risk for CRC. Individuals of under 50 years of age, with no specific risk factors, are considered to be at low risk for CRC, and do not require screening. However, individuals with personal and/or familial CRC risk factors should be considered for screening or observation, based on their personal background and medical history (colonic polyposis, Lynch syndrome, family history of CRC, ulcerative colitis, etc.) [15, 16].

**Table 4:** CRC screening recommendations in average risk populations

Recommendation	Evidence
Tests for the detection of hidden blood in faeces (SOH) are a useful tool, and must be considered in CRC screening programmes	A
Population screening programmes should include a quantitative SOHi (immunohistochemical) detection test with a positive cutoff point that guarantees optimal balance between sensitivity and specificity, based on available endoscopic resources	B
Opportunistic screening procedures should include SOHi detection tests, although a high-sensitivity SOHg (Guayaco) test could also be used	B
Flexible sigmoidoscopy is an efficient test that must be considered in CRC screening, at intervals of at least five years	B
Sigmoidoscopic detection of distal adenomatous polyps requires full colonoscopy (Grade A). This is not the case with hyperplastic polyps	B
A combined detection strategy using SOHg tests and flexible sigmoidoscopy should not be considered in CRC screening	B
Colonoscopies should be performed at intervals of at least 10 years	B
CT-scan colonoscopy should not be considered in CRC screening until more assessments are available on the benefits, costs and acceptability of the technique	B

- Tests for the detection of hidden blood in faeces (SOH) are a useful tool, and must be considered in CRC screening programmes (Grade A).
- Population screening programmes should include a quantitative SOHi (immunohistochemical) detection test with a positive cutoff point that guarantees optimal balance between sensitivity and specificity, based on available endoscopic resources (Grade B).
- Opportunistic screening procedures should include SOHi detection tests, although a high-sensitivity SOHg (Guayaco) test could also be used (Grade B).
- Flexible sigmoidoscopy is an efficient test that must be considered in CRC screening, at intervals of at least five years (Grade B).
- Sigmoidoscopic detection of distal adenomatous polyps requires full colonoscopy (Grade A). This is not the case with hyperplastic polyps (Grade B).
- A combined detection strategy using SOHg tests and flexible sigmoidoscopy should not be considered in CRC screening (Grade B).
- Colonoscopies should be performed at intervals of at least 10 years (Grade B).
- CT-scan colonoscopy should not be considered in CRC screening until more assessments are available on the benefits, costs and acceptability of the technique (Grade B).

### Recommendations for population-wide CRC [13, 16] screening programmes (Table 5)

- CRC screening should be offered to all individuals with no risk factors as from the age of 50. In accordance with guidelines established in our milieu (European Union, Spain and its regions), SOH detection tests should be performed every two years in men and women of 50–74 years of age (Grade A).
- Screening tests in population programmes should be based on a quantitative SOHi analysis with a positive cutoff point that guarantees an optimal balance between sensitivity and specificity, depending on the availability of colonoscopies. The choice of other screening tests (annual or biennial SOHg determination, sigmoidoscopy every 5 years or colonoscopy every 10 years) might be warranted if resources are available (Grade B).
- Individuals included in higher-risk groups (polyposis, etc.) must be identified, that they may benefit from specific screening and supervision measures (Grade A).

**Table 5:** Recommendations for population-wide CRC screening programmes

Recommendation	Evidence
CRC screening should be offered to all individuals with no risk factors as from the age of 50. In accordance with guidelines established in our milieu (European Union, Spain and its regions), SOH detection tests should be performed every 2 years in men and women of 50–74 years of age	A
Screening tests in population programmes should be based on a quantitative SOHi analysis with a positive cutoff point that guarantees an optimal balance between sensitivity and specificity, depending on the availability of colonoscopies. The choice of other screening tests (annual or biennial SOHg determination, sigmoidoscopy every 5 years or colonoscopy every 10 years) might be warranted if resources are available	B
Individuals included in higher-risk groups (polyposis, etc.) must be identified, that they may benefit from specific screening and supervision measures	A

## Lung cancer

According to the latest update of the GLOBOCAN 2012 study [17], lung cancer in Spain is the second most frequent cancer in men and the fourth most frequent cancer in women. Lung cancer is the third most frequent carcinoma if we combine the data for both sexes (26,700 cases in 2012, with a mortality rate of 21,118). This accounts for 20 % of all cancer deaths in Spain. Data for the US indicate that lung cancer is the main cause of early death from cancer in that country, with an estimated cost of 2.37 million years of life in 2010 [18].

These data reveal the importance of lung cancer prevention. If we bear in mind that the most important factor involved in the development of the disease—cigarette smoking—is clearly identified, neglect of preventive measures is inexcusable.

In recent years, different randomised studies [19, 20] have examined the potential value of low-dose computerised tomography in the prevention of lung cancer. These studies have revealed that the yearly performance of CT scans in high-risk groups (that is, individuals of 55–74 years of age who are active smokers or gave up the habit <15 years previously, with a history of at least 30 pack years) can decrease lung cancer mortality rates by 20 %, as compared to annual chest X-rays, with no added collateral damages resulting from radiological exposure [21].

Apart from the recommendation of yearly TC scans in high-risk groups [22], it is crucial to insist that smokers give up their habit, and to make sure that people who do not smoke do not begin to do so. Lung cancer screening should not be regarded as an alternative to giving up smoking (Table 6).

**Table 6:** Recommendations for lung cancer prevention

Recommendation	Evidence
Giving up smoking or not taking up the habit	A
Annual, low-dose CT scans in high-risk groups (individuals of 55–74 years of age who are active smokers or gave up the habit <15 years previously, with a history of at least 30 pack years)	A

Finally, it is essential that screening tests for lung cancer are personally discussed with all potential candidates, and carried out by multidisciplinary teams made up of experts in the management and treatment of the disease.

## Conclusions

Improved rates of cure for cancer are due in recent years to improvements in treatment and to the implementation of prevention programmes.

In some types of cancer, primary and secondary prevention measures can have a very significant effect on the reduction of incidence rates, and on the increase of survival as a result of earlier detection and diagnosis.

It is indispensable to encourage research into further prevention measures, especially in tumours whose rates of survival are the lowest.
